# Asthma and obesity: mechanisms and clinical implications

**DOI:** 10.1186/s40733-015-0001-7

**Published:** 2015-06-04

**Authors:** Cynthia Wilson Baffi, Daniel Efrain Winnica, Fernando Holguin

**Affiliations:** University of Pittsburgh Asthma Institute, 3459 Fifth Ave, NW 628, Pittsburgh, PA 15213 USA

**Keywords:** Obesity, Asthma, Obese asthma phenotype

## Abstract

Obesity is the most common asthma co-morbidity; it has been associated with increased risk for asthma exacerbations, worse respiratory symptoms and poor control. The exact mechanisms remain elusive and are probably multifactorial, stemming from mechanical alterations of the airways and lung parenchyma, to systemic and airway inflammatory and metabolic dysregulation that adversely influences lung function and or response to therapy. However, the fact that not every obese asthmatic is equally affected by weight gain highlights the many challenges and complexities in understanding this association. The factors that determine susceptibility may not depend on being obese alone, but rather the interactions with other phenotypical characteristics, such as age of asthma onset, gender and race to name a few. Inability to account for asthma phenotypes that are differentially affected by increasing body mass index (BMI) may contribute to the lack of consistent results across studies. This review will provide a succinct summary of obesity-related mechanisms and the clinical impact on asthma including highlights on recent progress.

## Introduction

Approximately 38 % of current adult asthmatics are also obese in the United States, and obesity has been shown to be risk factor for developing asthma. Obese asthmatics reports worse asthma control despite traditional asthma therapy, worse asthma-specific quality of life, and higher rates of healthcare utilization; however, the mechanisms driving this association remain unclear. Understanding the link between obesity and asthma may have major public health implications and has the potential to lead to development of future therapies. This review will give a concise examination of the association between asthma and obesity and provide conclusions on the current body of literature.

## Review

### The epidemiology of the obesity and asthma association

Obesity is associated with greater asthma morbidity and with increased prevalence and incidence rates. These associations have been inconsistently reported in males and females, across different races, and among all age groups. The heterogeneity of study results highlights the many complexities that characterize the obese – asthma association, including such issues as diagnostic bias, bi-directionality, differing asthma case definitions, and study designs. Despite these limitations, there is a general consensus from the existing literature that weight gain and obesity adversely affects the respiratory health of millions of subjects with and without asthma.

In a recent meta-analysis of 7 longitudinal cohort studies involving over 300,000 adults, there was a dose response effect between increasing BMI and the odds ratio (OR) of incident asthma. Compared to normal weight, a BMI ≥ 25 was associated with an OR of 1.5 (95 % CI; 1.2 – 1.6), whereas the OR for a BMI > 30 was 1.9 (95 % CI; 1.4 – 2.6), while similar ORs were observed in males and females [1]. Although these results strongly suggest a potential causal relationship, they were based on self-reported asthma and therefore more susceptible to diagnostic biases. In fact, when using stricter diagnostic criteria such as bronchial hyperresponsiveness, the obese – asthma association is less consistent, and obese subjects may be erroneously diagnosed as having asthma [2, 3]. Therefore, the extent by which greater BMI increases the likelihood of being diagnosed with asthma, independently of bias, remains unknown. A different question, however, is the role of obesity as a co-morbidity. As it has been shown in multiple cross sectional studies, increasing body mass index (BMI) strongly influences asthma control and severity [3]. Even when fully compliant and in accordance with treatment guidelines, having a BMI above normal diminished the probability of achieving adequate symptom control. Obesity is also linked to more frequent and severe respiratory symptoms. In the Centers for Disease Control (CDC) four state (Alabama, California, Texas, and Illinois) sample of the National Asthma Survey, those with a BMI > 30 had more daily symptoms, used rescue inhalers more frequently, and were less likely to achieve remission [4]. Given that on average obese asthmatics, both children and adults, are more symptomatic and poorly controlled, it is not surprising that studies have shown them to have increased rates of healthcare utilization and greater healthcare expenditure [5, 6], though this has not been uniformly shown across studies [7].

### Are obese asthmatics a unique clinical phenotype?

An important unanswered question is whether being obese and having asthma constitutes a unique clinical phenotype; in other words, does the interaction of obesity (as an environmental risk factor) with underlying genetic traits (or other host susceptibility factors) lead to a set of defined observable traits? If this were correct, all asthmatics with a BMI > 30 would share unique clinical characteristics; however, given that patients with different clinical asthma phenotypes can be obese, and not every asthmatic seems to be equally impacted by weight gain, this idea seems rather simplistic. To further illustrate this point, in the Severe Asthma Research Program (SARP) cluster study, the proportion of obesity across the 5 asthma phenotype clusters ranged from 24 – 51 %, and it was greater than a third of the population in all of the clusters except for cluster 1 [8]. However, clinical characteristics associated with the highest BMI cluster that have been consistently described across studies include having late onset asthma, lower exhaled nitric oxide (eNO) levels, less airway eosinophils, and reduced atopy [8–10]. Asthmatics in this cluster are disproportionally symptomatic for the level of airway inflammation or functional impairment and have a high rate of controller medication usage and healthcare utilization. Unfortunately, given the cross sectional comparison between cluster groups, it is unknown whether obesity antecedes the development of these phenotypical characteristics or whether subjects in this cluster are more likely to gain weight. This leads to another unanswered question; is obesity a risk factor for or a consequence of increased asthma severity? The response may depend on how it relates to additional phenotypical factors. For example, in a cross sectional study of the SARP study population, the relationship between BMI and duration of asthma (in years) varied with regard to whether the onset of asthma was early or late (before or after 12 years of age) [5]. In the early onset category, independent of other confounders, BMI increased linearly for every year of having asthma since diagnosis; in contrast, there was no association in the late onset group. One possible interpretation is that in contrast to the later onset asthma, early onset is a potential risk factor for weight gain. However, it remains to be determined whether obesity that antecedes or occurs after an asthma diagnosis impacts asthma severity or control any differently.

### Impact of obesity on asthma control

Different anthropometric measures, including BMI or other adiposity indexes, have been shown to be significant determinants of asthma control for adults in some studies, even when adjusting for other confounders [11–13]. In contrast, other studies have failed to show this trend. For example, in a predominantly African American adult, inner city asthma population, increasing BMI was not shown to be independently associated with decrements in the asthma control questionnaire (ACQ) or asthma control test (ACT) [7]. Although on average this study population was poorly controlled and had a high healthcare utilization rate, there were no associated trends with increasing BMI. In a large study of 2,174 children participating in the Genes-environments and Admixture in Latino Americans (GALA II) Study and the Study of African Americans, Asthma, Genes, and Environments (SAGE II), obesity increased the odds of being poorly controlled (defined by the American Thoracic Society guidelines) by 33 % in boys. Interestingly, this association was modified by ethnicity and race in girls, in whom obesity increased the OR for poor control in Latinos, but not in African Americans [14].

Overall, increasing BMI can adversely influence asthma control; however, the direction and magnitude of this association will depend on how the study population is constituted, specifically, the proportions of different asthma phenotypes (i.e. race, age of onset, and gender), some of which may be more or less affected by having greater adiposity or BMI.

### Potential mechanisms

There are multiple mechanisms by which obesity can potentially worsen asthma, including its effects on pulmonary physiology and mechanics. Increasing weight gain can have profound implications on lung physiology, including the development of restriction from greater adiposity around the chest wall and abdomen. This can result in reduced total lung capacity and most notably, low expiratory reserve volume, from upward diaphragmatic displacement due to increased abdominal fat; consequently, airway closure occurs at or above functional residual capacity in the dependent lung zones, which can lead to significant ventilation/perfusion mismatching [15]. Although obesity is not associated with more airway obstruction, some studies have found it to be a risk factor for greater bronchial hyperresponsiveness [3]. In the Veterans Health Normative Aging Study, having a BMI > 29 was associated with a ten-fold increase in odds for developing methacholine responsiveness [16]. Whether or not asthma and obesity synergistically increase bronchial hyperresponsiveness (BHR) has not been consistently shown [3]; however, there is evidence that both conditions jointly act to impair the degree of bronchodilation after a deep inhalation (DI), which is a mechanical airway dysfunction that in asthmatics has been associated with increased indices of airway inflammation (See Table 1) [17–19].

**Table 1:** Implications of obesity and the relationship with asthma. Obese asthmatics have multiple consequences related to excess adipose tissue, including mechanical or physiologic effects on lung function and the airways as well as changes in the immune response and metabolic effects. The combination of these alterations contributes to the phenotypic characteristics of the obese asthmatic

Mechanical or physiologic effects	Lung function	• Restriction or reduced total lung capacity and decreased expiratory reserve volume
		• Ventilation and perfusion mismatch
	Airways Changes	• Bronchial hyperresponsiveness
		• Loss of beep breath induced bronchodilation
		• Reduced exhaled NO (certain phenotypes)
Immune and metabolic effects	Immune function	• Decreased airway eosinophils (lumen, sputum)
		• Increased airway neutrophils
		• Predominately Th-1 related inflammation versus Th-2
		• Potential IL-17 related inflammation
		• Enhanced inflammatory/oxidative response to elevated leptin levels
	Metabolic function	• Higher plasma and airway leptin levels with reduced airway leptin receptors
		• Leptin receptors in visceral fat and relationship with bronchial hyperresponsiveness
		• Leptin may increase oxidative stress levels
		• Effect of adiponectin remains unclear
		• Lower L-arginine/ADMA ratio and increase in oxidative stress resulting in an impaired bronchial dilatory response

**Fig. 1:**
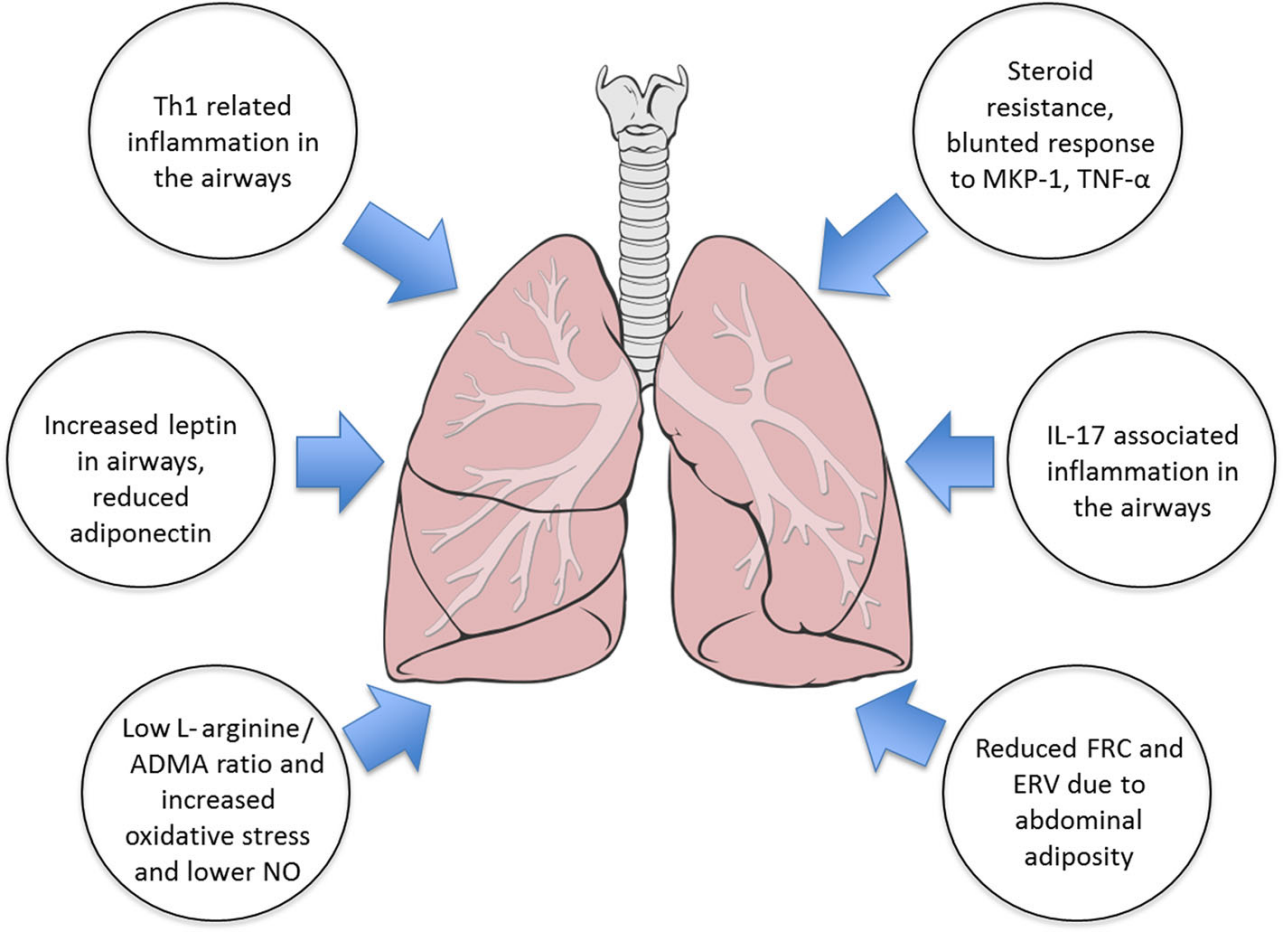
Mechanisms of Obese Asthma. A variety of mechanisms have been proposed as drivers of the physiologic and clinical observations in obese asthmatics, including changes in adipokines; T-helper type 1 (Th-1) skewed airway inflammation; lower asymmetric dimethylarginine (ADMA) to L-arginine ratio resulting in increased oxidative stress and decreased physiologic nitric oxide (NO), a mediator in smooth muscle dilatation; reduced functional residual capacity and expiratory reserved volume due to excess abdominal adiposity; interleukin-17 (IL-17) associated airway inflammation; steroid resistance and dampened response to mitogen-activated protein (MAP) kinase phosphatase-1 (MKP-1)

Yet the fact that not every obese asthmatic experiences an increase in asthma severity suggests that other non-mechanical factors are also involved. Broadly speaking, it has been previously proposed that obesity, as a chronic systemic inflammatory disorder, could affect asthma by enhancing airway inflammation. However, clinical and epidemiological studies have shown that this assertion is far more complicated and unlikely to be explained by an interaction between classical markers of systemic and airway inflammation [20]. In cross-sectional studies, it has been shown that with increasing BMI there is either no change or a reduction in the percentage of airway eosinophils [21, 22]. Indeed, analysis of nearly 1000 patients undergoing repeated sputum samples from the Asthma Clinical Research Networks, showed that compared to the overweight and lean categories, the obese category had the largest proportion of non-eosinophilic asthmatics [23]. However, this phenomenon may not necessarily reflect an absolute reduction of airway eosinophils in obesity, but rather, reduced migration into the airway lumen. This observation was shown in a recent study of severe asthmatics by Desai et al, in which obesity was associated with increased sub-mucosal eosinophils (yet not in the airway lumen) and with greater IL-5 sputum levels [24]. This phenomenon in obese asthmatics may be due to altered eosinophil survival or clearance or possibly a functional change in eosinophil response to cytokines or chemokines. In contrast, obesity has been associated with increased airway neutrophilia [25]. Given these results, it would appear that the obesity-mediated changes in airway inflammation are more consistent with a non-predominant Th-2 phenotype, and potentially a more Th-1 polarized immune response [26]. Immune innate cells that produce IL-17 have been implicated in murine obese models and detected in human bronchoalveolar lavage fluid (BAL) and may constitute yet another non-Th2 pathway [27]. With increasing adiposity, there is a corresponding increase in leptin and a reduction in adiponectin. Both of these changes have been implicated in the obese – asthma pathogenesis [28]. Leptin is readily detected in the airways, is higher among asthmatics, and increases in relation to plasma leptin levels and BMI [29, 30]. Human airway epithelial cells express leptin receptors, which are reduced with increased asthma severity, and are associated with airway remodeling changes [31]. Peripherally, the magnitude of leptin receptor expression in visceral fat has been related to BHR, which has led to the concept that obesity-asthma is potentially a disease of peripheral adipose tissues [32]. *Ex vivo*, leptin has been shown to increase the oxidative and inflammatory response of alveolar macrophages derived from overweight and obese asthmatics [30]; however, whether or not increased airway leptin is associated with greater BHR, airway oxidative stress, or a diminished response to steroids is not known. Adiponectin, on the other hand, is thought to have protective effects. It can also be found in the airways, yet it does not closely relate to BMI or plasma levels [29]. In females, higher plasma adiponectin levels are associated with decreased asthma risk [33], yet whether it has any anti-inflammatory or immune modulator effects in the human airway is not clear; or whether its plasma or airway levels are related to poor asthma control, is not known (See Table 1 and Fig. 1).

### Oxidative stress and metabolic dysregulation

It is widely known that both asthma and obesity are chronic diseases characterized by greater oxidative stress; however, it is not known whether this is synergistically increased by both conditions. Because the majority of research has been related to inflammation and immunity, a potential role for metabolic derangements, as a source of oxidative stress, has been greatly overlooked. With this in mind, one potential area that may have particular relevance to the obese-asthma association is related to changes in nitric oxide (NO) metabolism. There is an inverse linear association between exhaled NO (eNO) with increasing BMI in late onset (after childhood) asthmatics compared to early onset [34]. Interestingly, this association has also been described in children [35]. This inverse association may be secondary to an imbalance between L-arginine, the precursor of NO and substrate for inducible nitric oxide synthase (iNOS), and asymmetric di-methyl arginine (ADMA), which is an endogenous inhibitor of all NOS enzymes that produce NO [34]. Lower L-arginine might result from increased arginase activity, which is associated with asthma severity, and increased ADMA has been related to obesity and metabolic syndrome [36, 37]. As a result of having lower L-arginine/ADMA ratios, obese asthmatics produce less NO and more anion superoxide from iNOS uncoupling in which electrons are shifted to molecular oxygen rather than toward production of NO [38]. Having lower airway NO bioavailability at baseline may impair the degree of physiological bronchial dilation, leading to increased respiratory symptoms [39]. Indeed, increased airway and systemic ADMA is inversely associated with eNO [40] and in the SARP study population, with more frequent respiratory symptoms, poorer quality of asthma and lower lung function [34].

#### Steroid resistance

Post hoc analyses of clinical trials have shown that the response to inhaled steroids is different across BMI categories [41, 42]. Compared to lean and overweight categories, obese and morbidly obese subjects have a reduced percentage change in FEV_1_ after starting inhaled corticosteroids (ICS); moreover, there is an inverse association between the percent of asthma control days (ACD) and BMI, among subjects randomized to ICS, leukotriene blockers, or placebo [43]. Although it appeared that the effect of BMI on ACD was less for subjects randomized to the leukotriene arm, subsequent studies have shown that response to this medication is not modified by BMI categories. The reduced steroid response in obese asthmatics could be secondary to increased resistance; as shown by Sutherland et al, who showed that being obese was associated with an *in vitro* blunted response to dexamethasone-induced mitogen-activated protein (MAP) kinase phosphatase-1 (MKP-1) and baseline tumor necrosis factor (TNF)-alpha in peripheral blood mononuclear cells (PBMCs) and bronchoalveolar lavage cells.

Obesity – mediated steroid resistance could also be secondary to low vitamin D levels, which are known to be inversely related to BMI and associated with increased asthma morbidity [44]. Early studies in asthmatic children and adults suggested that vitamin D deficiency is associated with lung function impairment, worse AHR, more severe asthma, and decreased response to corticosteroid therapy [44–46]. However, in the recent National Institutes of Health (NIH) AsthmaNet Clinical Trial of Vitamin D Supplementation in Asthma (VIDA), vitamin D was not more effective on obese asthmatics. Specifically, the VIDA study, a randomized, double-blind, parallel, placebo-controlled trial sought to determine if vitamin supplementation in asthmatics using inhaled corticosteroids improved asthma outcomes with a primary outcome of time to first asthma treatment failure (based on decline in lung function and increased use of beta-agonists or systemic corticosteroids, and increased healthcare utilization). In this study, when comparing those asthmatics with BMI < 25 versus BMI ≥ 25, vitamin D supplementation did not show a reduction in asthma treatment failures in the obese group [47].

Ultimately, increased steroid resistance may explain why, even when adequately treated, asthmatics – regardless of severity - with a BMI > 25 are less likely to transition from an uncontrolled state to a controlled state over time [48]. The longitudinal SARP study, currently underway, which is a multicenter observational study of moderate and severe asthmatics, will provide important information as to how BMI influences the response to systemic steroids in children and adults with asthma [49].

### Co-morbidities (obstructive sleep apnea, gastroesophageal reflux disease, and metabolic syndrome)

Becoming obese increases the risk for other chronic illnesses, which are also linked to increased risk of asthma or worsened respiratory symptoms. Data from the American Lung Association Clinical Trials Network showed in a *post hoc* analysis involving 402 patients, that obstructive sleep apnea (OSA), but not gastroesophageal reflux disease (GERD), was associated with poor asthma control. Other studies have also shown that OSA can contribute to asthma control and severity [50, 51]. Together, these data make a strong case for OSA as a potential mechanistic pathway for obesity and greater asthma morbidity in some patients. Depression is another co-morbidity that is more common in obese subjects, is independently associated with poor control, and in one study, it has been shown to mediate symptoms between increased BMI and asthma [52, 53].

An important confounder to the obese – asthma association is metabolic syndrome (MetSyn), which occurs in ~20 % of the general population and 60 % of obese subjects. It is defined as having at least 3 out of 5 of the following: a) glucose intolerance, b) hypertension, c) abdominal obesity, d) dyslipidemia – low high density lipoprotein (HDL), and hypertriglyceridemia [54]. The MetSyn has been related to asthma independently of BMI; in the Nord-Trøndelag Health Study (HUNT) study, a large European cohort study, MetSyn diagnosis was associated with incident asthma after adjusting for BMI [55]. However, whether the obese – asthma relation is explained by MetSyn remains controversial. In a large study of 4,619 eligible participants in the Coronary Artery Risk Development in Young Adults (CARDIA) cohort followed over 25 years, MetSyn predicted asthma incidence in women, but this association was found to be mostly confounded by BMI [56].

### Treating the obese asthmatic

While there are no pharmacologic strategies to specifically treat obese asthmatics, weight loss interventions, both surgical and nutritional, have been tested and shown to have varying degrees of effectiveness in improving the respiratory health of these patients. The BMI reduction in one year following bariatric surgery has been shown to significantly improve average ACQ and ACT scores [57, 58]. However, in one study, BHR improved only among patients with low or normal IgE levels, suggesting that different phenotypes may differentially benefit from weight loss [58]. In a randomized clinical trial for children with asthma, a short term dietary intervention based on caloric restriction significantly improved asthma control when compared to the control group; ACQ improved significantly in the diet intervention group when compared to the wait listed control group (-0.4 [-0.7, 0.0] vs. 0.1 [0.0, 0.6], *P* = 0.004) [59]. According to a Cochrane meta-analysis, weight loss interventions can improve outcomes in overweight or obese asthmatics, however, the majority of studies suffered from severe design flaws [60]. In a recent study this year, 330 obese adults with uncontrolled asthma were randomized to usual care versus usual care plus a 12-month intervention targeted at weight loss and increased physical activity for asthma control. The primary outcome was change in ACQ from baseline to 12 months. This study showed that weight loss of at least 10 % or greater was associated with improvement in ACQ and clinical improvement in asthma [61]. An important question, which remains to be answered, is whether weight loss improves response to inhaled or systemic corticosteroids.

## Conclusions

- Obesity may be a risk factor for increased asthma severity and poor control in a subgroup, but not all patients
- It is unlikely that there is one unique obese – asthma phenotype
- The relationship between obesity and asthma is bi-directional
- Patient susceptibility to obesity-mediated effects on asthma may depend on the interaction with other demographic and clinical factors, such as gender, race, age of asthma onset, and atopy.
- There are multiple potential mechanisms yet there is not a unified consensus as to which are more clinically relevant or how they may work in parallel.
- Weight loss is the only available specific intervention to treat the effects of obesity on asthma. The effectiveness of this intervention may depend on other phenotypical factors and the degree of weight loss.
- Studies involving weight loss should be undertaken to test the clinical and biological relevance of proposed mechanistic pathways between obesity and asthma.

## Abbreviations

BMI: Body mass index
OR: Odds ratio
CDC: Centers for Disease Control
SARP: Severe asthma research program
eNO: Exhaled nitric oxide
BHR: Bronchial hyperresponsiveness
DI: Deep inhalation
ACQ: Asthma control questionnaire
ACT: Asthma control test
GALA: Genes-environments and admixture in Latino Americans
SAGE: Study of African Americans, asthma, genes, and environments
Th1: T-helper type 1
IL-17: Interleukin-17
BAL: Bronchoalveolar lavage
Th2: T-helper type 2
NO: Nitric oxide
iNOS: Inducible nitric oxide synthase
ADMA: Asymmetric dimethylarginine
FEV_1_: Forced exhaled volume in 1 second
ICS: Inhaled corticosteroids
ACD: Asthma control days
MAP: Mitogen activated protein
MKP-1: Kinase phosphatase-1
TNF: Tumor necrosis factor
PBMCs: Peripheral blood mononuclear cells
NIH: National Institutes of Health
VIDA: Vitamin D supplementation in asthma
OSA: Obstructive sleep apnea
GERD: Gastroesophageal reflux
MetSyn: Metabolic syndrome
HDL: High density lipoprotein
HUNT: Nord-Trøndelag health study
CARDIA: Coronary artery risk development in young adults
IgE: Immunoglobulin E
